# Hearing health professionals’ attitudes and perceived skills toward artificial intelligence

**DOI:** 10.1186/s12909-025-08505-9

**Published:** 2026-01-10

**Authors:** Joanie Ferland, Matthieu J. Guitton, Andreanne Sharp

**Affiliations:** 1 Faculty of Medicine, Université Laval, Quebec City, QC Canada; 2 CERVO Brain Research Center, Quebec City, QC Canada

**Keywords:** AI, Artificial intelligence, Audiologist, Hearing-aid acousticians, Self-efficacy, Training needs

## Abstract

**Background:**

As artificial intelligence becomes increasingly integrated into healthcare, understanding health professionals’ perceptions and comfort with these technologies is gaining importance. This study investigated hearing health professionals’ eHealth literacy, perceptions of artificial intelligence (AI), and AI self-efficacy, examining how these constructs vary across professions and relate to use of AI. It also explored their interrelationships and documented training needs and preferred providers of that training to support the integration of AI into clinical practice.

**Methods:**

An online survey was conducted among hearing health professionals (audiologists and hearing-aid acousticians) in the province of Quebec, Canada. It included validated instruments to assess eHealth literacy (eHEALS), perceptions of AI (SHAIP), and AI self-efficacy (AISES and an adapted RUSH scale). Participants also reported their use of AI (personal and professional), sociodemographic and professional characteristics, and their AI training needs, including preferred types of organizations to deliver that training.

**Results:**

Data from 114 professionals (mean age 38.1 ± 1 years; 75.5% women) showed that eHealth literacy scores were similar across professions and between AI users and non-users. Perceptions of AI were more positive among hearing-aid acousticians and among participants using AI, regardless of their profession. While AI self-efficacy did not differ by profession, scores were higher among AI users. All participants recognized the need for AI training, with, surprisingly, professional orders and the corporate sector more often identified as preferred providers than post-secondary institutions.

**Conclusions:**

This study revealed that hearing health professionals who had already adopted AI into their practice showed more positive perceptions of AI and better AI self-efficacy. AI training, and those providing it, should consider professionals’ current attitudes and perceived skills towards AI to facilitate its integration into clinical practice.

**Supplementary Information:**

The online version contains supplementary material available at 10.1186/s12909-025-08505-9.

## Background

In the last few years, technologies related to or incorporating artificial intelligence (AI) have emerged as major transformative agents. AI-driven applications impact all aspects of our daily lives, and even spawn within the medical field. The benefits of using AI in healthcare settings are numerous, and the use of AI has been seen as a potential solution to address significant health-related challenges, such as rising healthcare costs, global shortages of healthcare workers, lack of accessibility for underserved communities and enhance diagnostic accuracy and treatment outcomes [1]. Spearheaded by their use in radiology, AI tools have been developed and proposed to assist health professionals in decision-making or diagnosis for various health conditions, or even as an auxiliary under the form of a chatbot [2–7]. Therefore, the integration of AI has become increasingly prevalent in various medical sectors.

In the field of hearing health, AI has already been used for multiple applications [8], including but not limited to diagnosis assistance for ear disease [9–11] or vestibular pathologies [12–13], otological imagery analysis [14–15], or machine learning-based prognostic of surgical outcomes based on radiological data [16–17]. AI-based applications are also being increasingly used for hearing assessments [18–20]. Furthermore, in the context of hearing health, AI finds a major potential when integrated with or within hearing aids, as deep learning technologies could allow significant enhancement of the current signal-processing algorithms’ efficacy [21].

As artificial intelligence becomes increasingly integrated into healthcare, understanding health professionals’ perceptions and comfort with these technologies is gaining importance. Previous studies have explored attitudes toward AI among various groups, including general practitioners [22], medical specialists [23], and other healthcare providers [24]. However, research focusing specifically on hearing health professionals, such as audiologists and hearing-aid acousticians, remains limited, despite their growing exposure to AI-based tools in clinical practice. A population of general practitioners, ENT specialists, and audiologists from the United Kingdom expressed both excitement towards the integration of AI in their practice as well as openness to its use for clinical decisions or diagnosis and concerns regarding reduced learning opportunities for clinicians, diagnostic bias, legal liability and data misuse [25]. Despite a very small sample size (*n* = 5), another study exploring health professionals’ attitudes toward AI in Australia suggested that audiologists were comparable to other health professions regarding their perception of AI, but could potentially feel more prepared for the use of AI in their work [26]. Finally, a survey conducted in India among audiologists and speech-language pathologists revealed diverse views on the integration of AI into clinical practice. While many acknowledged its potential to support diagnosis and treatment planning, others expressed uncertainty or concern about its implications for patient care and professional roles [27]. While these studies provide valuable insights into perceptions of AI, none have directly assessed professionals’ eHealth literacy skills or their perceived self-efficacy in using AI which are variables that have previously shown that could influence AI adoption.

Research has shown that higher eHealth literacy—the ability to seek, understand, and apply digital health information, is associated with greater openness to innovation and more effective use of digital health tools, including those using AI [28–30]. It is therefore reasonable to hypothesize that eHealth literacy may influence how hearing health professionals integrate AI into their clinical practice. Similarly, AI self-efficacy, defined as confidence in one’s ability to understand and use AI technologies, is a well-established predictor of technology adoption. According to Bandura’s social cognitive theory (1997), self-efficacy affects not only the decision to engage with new technologies but also persistence in overcoming challenges. In healthcare, professionals with higher self-efficacy are more likely to adopt and effectively use AI-based systems [31]. Despite their relevance, these constructs remain underexplored in hearing healthcare. Investigating them could offer a more comprehensive understanding of the factors influencing AI adoption and how they relate to professionals’ perceptions of AI. Based on a comprehensive survey conducted among hearing health professionals in the province of Quebec (Canada), this study aimed to examine three key constructs: eHealth literacy, perceptions of AI, and perceived AI self-efficacy, the latter serving as a proxy for professionals’ comfort with AI-based technologies. Specifically, the primary objective was to explore whether these variables differed according to profession (audiologist, hearing-aid acoustician), professional use of AI (yes, no), or the interaction between the two. To support this analysis, we first described participants’ sociodemographic and professional characteristics to better understand potential differences across groups. Additionally, we also investigated the relationships among eHealth literacy, AI perceptions, and AI self-efficacy. Finally, we assessed professionals’ self-reported training needs and preferred sources of AI training, with the goal of informing strategies for the broader and more harmonious integration of AI into hearing healthcare practice.

## Method

### Participants

A cross-sectional study design was used to survey audiologists and hearing-aid acousticians. Participants were hearing health professionals (audiologists, hearing-aid acousticians) from the province of Quebec (Canada). As of March 2024, the population of audiologists and hearing-aid acousticians in the province was 546 and 545 members respectively [32–33]. Each profession has distinct reserved acts, reflecting differences in their roles and responsibilities. The role of the audiologist is “assess the auditory functions, determine a treatment and intervention plan and ensure its implementation in order to improve or restore communication for a person in interaction with his environment” [34]. Audiologists also evaluate vestibular functions and may recommend compensatory technologies (such as hearing aids, cochlear implants, or assistive listening devices) when appropriate. As part of the audiological intervention, they may also adjust these devices, particularly when working in a rehabilitation center. The role of hearing-aid acousticians is to “sell, fit, adjust or replace a hearing aid” [35]. However, they can’t perform these acts if they are not prescribed by a physician, speech therapist or audiologist attesting the necessity of a hearing aid. Prospective participants were solicited through ads published on local hearing care professionals’ social media groups. Links to the survey were also sent via email and posted on professional associations and orders’ (regulatory organizations) websites. Questionnaires were filled out voluntarily and anonymously by hearing health professionals from October 20th 2024 to May 30th 2025.

### Survey design

The survey was elaborated using the Guidelines for Reporting Results of Internet E-Surveys [36]. The survey was built and administered using Gorilla Experiment Builder (https://gorilla.sc/), an online platform known for its questionnaire-building functionality. The final questionnaire consisted of 63 items, divided into six sections: (1) sociodemographic information; (2) use of AI and training needs; (3) eHealth literacy scale; (4) AI perception tool; (5) self-efficacy in healthcare work; (6) AI self-efficacy scale (Appendix A).

#### Sociodemographic questionnaire

Sociodemographic and professional characteristics were gathered through 12 questions. Participants were asked general questions about their age, gender, native language, profession (audiologists, hearing-aid acousticians), age at graduation, and professional experience (in years) as a hearing health professional. Of note, the participants’ biological sexes were not included in the sociodemographic questionnaire, as gender was found to be a more adequate data in this population, in part due to the data available by the regulatory bodies (professional orders) that only identify professionals’ gender. Additional details were gathered regarding their specificities of professional clinical practice, including membership to a professional order, location of clinical practice (urban, rural, or both), work setting (private practice, hospital/public clinic, rehabilitation center, industry, teaching, administrative, other), type of clientele (pediatric (0–5), pediatric (6–18), adult, older adult), field of practice (pathologies (tinnitus, eustachian tube dysfunction, hyperacusis/misophonia, deaf blindness, noise-induced hearing loss, auditory processing disorder, vestibular disorders), assistive and protective hearing devices (assistive listening devices, molded earplugs, cochlear implants, bone-anchored hearing aids), professional acts (auditory training, cerumen removal, communication strategies, hearing aid recommendation, hearing aid sale, hearing aid fitting/adjustments, educational audiology), assessment (otoacoustic emissions, auditory evoked potentials), and hearing screening), and adoption of telepractice (yes, no).

#### Use of AI and training needs

Participants were asked about their use of AI in both personal and professional contexts. Personal use was assessed with a multiple-choice question (yes, no, uncertain), while professional use and perceived value of AI in clinical settings were explored through an open-ended question. An additional open-ended item explored participants’ perceived need for further training on the use of AI in their clinical practice. Finally, participants were asked to select, from a multiple-choice list, which institutions they believe should be responsible for providing such professional training (post-secondary education (college, university), professional orders, professional associations, manufacturers (hearing aids, implants), equipment manufacturers, hearing health clinics, information technology companies, self-learning through Internet or self-learning through manuals).

#### eHealth literacy skills

The participants’ eHealth literacy skills were assessed using the French version of the eHeatlh Literacy Scale (F-eHEALS), a 10-item validated questionnaire (5-point Likert scale, ranging from *strongly disagree* to *strongly agree*) [37–38]. The final score was calculated using only the last eight items of the eHEALS, as the first two items are considered supplementary and are intended to assess general interest in using eHealth technologies [37]. These items are not part of the validated scale. Furthermore, limiting the analysis to the eight core items is consistent with previous studies, thereby facilitating comparisons of healthcare professionals’ eHealth literacy with findings reported in the existing literature. The final score reflects the users’ knowledge, comfort and perceived skills in finding and using health information online [38]. A higher score on the scale corresponds to a higher degree of eHealth literacy.

#### Perceptions of artificial intelligence

The participants’ perceptions of artificial intelligence were evaluated with a French translation of the 8-item version of the Shinners Artificial Intelligence Perception Tool (SHAIP) (5-point Likert scale, ranging from *strongly disagree* to *strongly agree*) [39–40]. Items were grouped into two dimensions: *perception of professional impact* (five questions), and *perception of preparedness for AI* (three questions) [41]. A higher score corresponds to healthcare professionals’ favorable perception of artificial intelligence.

#### General AI self-efficacy

General AI self-efficacy was evaluated through a French translation of the 22-item questionnaire Artificial Intelligence Self-Efficacy Scale (AISES) (7-point Likert scale, ranging from *strongly disagree* to *strongly agree*) [42]. Questions were grouped into four dimensions: *assistance* (perceived helpfulness or value of AI technologies/products for the task), *anthropomorphic interaction* (the degree of anthropomorphism individuals perceive when interacting with AI technologies/products), *comfort with AI* (an individual’s emotional awareness when interacting with AI technologies/products), and *technological skills* (an individual’s background knowledge and level of confidence in using AI technologies/products). Normative scores were established by the original authors on a sample general adult population with internet access, particularly individuals familiar with or exposed to AI applications (*n* = 314), with 10th percentile scores at 87.00, 50th percentile at 109.00 and 90th percentile at 136.00 [42].

#### AI self-efficacy in a healthcare professional setting

The participants’ AI self-efficacy in their work context was assessed using a French translation of an adapted version of the Robot Use Self-Efficacy in Healthcare work (adapted RUSH). In this 6-item questionnaire (5-point Likert scale, ranging from *totally disagree* to *totally agree*), “care robots” was replaced by “artificial intelligence” to reflect the topic of the present study. The original questionnaire generates two scores, namely the RUSH-6 (a 6-item analysis) and the RUSH-3 (a subset of 3 items) [43]. For both versions, a higher scores correspond to higher degrees of AI self-efficacy at work in a healthcare professional context.

### Statistical analyses

All data were extracted from the online platform Gorilla Exp builder (https://gorilla.sc/), and analyzed using IBM SPSS Statistics (version 30), R Studio (version 2025.05.1 + 513) and Jamovi (version 2.5.4.0) (https://www.jamovi.org) running on MacOS Sequoia 15.0. Data extracted from the qualitative sections of the questionnaire were transformed into categorical variables for analysis following an inductive method. Specifically, responses regarding professional use of AI were recoded into a binary variable (yes, no), while responses concerning the perceived need for AI training were analyzed and categorized into four options (yes, no, in the future, other). Additionally, for professionals using AI in a clinical setting, open-ended responses on how they used AI were coded. These codes were then grouped into eight categories of AI applications: integration in hearing aids and programming, text composition and editing, content creation and editing, research and summary tool, hearing aid manufacturers’ mobile applications, data logging for hearing aid users, translation, and voice transcription. Concerning the variable personal use of AI, participants who selected the “uncertain” option (*n* = 13) were excluded from comparative analyses, as this response reflects ambiguity in self-assessment, which may result from limited exposure to AI, misunderstanding of the concept, or indecision. Data on age and professional experience were also computed into categorical variables for comparative analyses, with age categorized into five groups of even time intervals (10 years) and professional experience categorized into six groups of even time intervals (5 years). Gender was classified as a dichotomous categorical variable based on participants’ selection of either “woman” or “man” from a multiple-choice question. Although additional gender identities were offered, only these two options were selected by participants. For questionnaires that included only a global score reflecting the measured variable, this score was used for analysis. In contrast, for questionnaires that contained relevant subscale scores, these were computed separately and incorporated into the analysis using a factorial approach. All descriptive statistics are presented using mean values and their corresponding standard errors (SEM).

#### Sociodemographic characteristics comparisons

Mann-Whitney U tests were used to compare age and professional experience between professions. Fisher’s exact tests were used to assess associations between profession and categorical variables such as gender, telepractice adoption, fields of practice, and both personal and professional use of AI. Chi-square goodness-of-fit tests were used to compare each profession’s gender distribution to provincial benchmarks reported by their respective professional orders [32–33]. Chi-square tests of independence were conducted to examine the association between telepractice adoption and clinical practice location. Fisher’s exact tests were used to assess the association between telepractice adoption and both personal and professional use of AI.

#### Questionnaire outcomes across professions

Questionnaire scores were computed for all 114 participants, with the exception of the variable *professional use of AI*, where 112 responses were computed due to two participants not filling out this section of the questionnaire. The association between eHealth literacy scores (F-eHEALS), professions, professional use of AI, and their interaction was analyzed using a non-parametric Aligned Rank Transform Analysis of Variance (ART ANOVA), as the data for this variable were not normally distributed. A multivariate analysis of variance (MANOVA) was conducted to examine the effects of profession, professional use of AI, and their interaction on participants’ perceptions of AI, as assessed by two sub-scores from the SHAIP scale: perceived professional impact and perceived preparedness for AI. AI self-efficacy in a healthcare setting, measured using the adapted RUSH-6 (global score) and adapted RUSH-3 (subset score), was analyzed using two separate ANOVAs. These analyses tested whether participants’ scores differed based on profession, professional use of AI, and their interaction. A MANOVA was conducted to examine the effects of profession, professional use of AI, and their interaction on participants’ general AI self-efficacy (AISES) scores, assessed across four continuous dimensions: assistance, anthropomorphic interaction, comfort with AI, and technological skills. Additional comparative analyses were performed to examine differences in each construct across sociodemographic and professional characteristics. Student’s t-tests were conducted to compare perceptions of AI (SHAIP) and AI self-efficacy in a healthcare professional setting (adapted RUSH-6) according to personal use of AI, but also to compare perceptions of AI (SHAIP), general AI self-efficacy (AISES) and AI self-efficacy in a healthcare professional setting (adapted RUSH-6) according to adoption of telepractice. Mann-Whitney U tests were used to compare eHealth literacy (F-eHEALS), general AI self-efficacy (AISES) and AI self-efficacy in a healthcare professional setting (adapted RUSH-3) according to personal use of AI, but also to compare eHealth literacy (F-eHEALS) and AI self-efficacy in a healthcare professional setting (adapted RUSH-3) according to adoption of telepractice. ANOVAs were conducted to determine whether : (1) perceptions of AI (SHAIP), general AI self-efficacy (AISES) and AI self-efficacy in a healthcare professional setting (adapted RUSH-6 and adapted RUSH-3) differed across age (5 levels), (2) perceptions of AI (SHAIP), general AI self-efficacy (AISES) and AI self-efficacy in a healthcare professional setting (adapted RUSH-6 and adapted RUSH-3) differed across professional experience (6 levels), (3) perceptions of AI (SHAIP), general AI self-efficacy (AISES) and AI self-efficacy in a healthcare professional setting (adapted RUSH-6) differed across location of clinical practice (3 levels). Finally, Kruskal-Wallis tests were conducted to determine whether scores of eHealth literacy (F-eHEALS) differed across age (5 levels), professional experience (6 levels), but also to determine whether scores of eHealth literacy (F-eHEALS) and AI self-efficacy in a healthcare professional setting (adapted RUSH-3) according to location of clinical practice (3 levels). Assumptions underlying parametric analyses were verified prior to all tests.

#### Correlations across outcome measures

Spearman correlations were used to investigate relationships between eHealth literacy (F-eHEALS), perceptions of AI (SHAIP), general AI self-efficacy (AISES) and AI self-efficacy in a healthcare professional setting (adapted RUSH-6).

#### Influence of gender on outcome measures

Student t tests were used to compare perceptions of AI (SHAIP), general AI self-efficacy (AISES) and AI self-efficacy in a healthcare professional setting (adapted RUSH-6) according to gender. Mann-Whitney U tests were used to compare eHealth literacy skills (F-eHEALS) and the subset of AI self-efficacy in a healthcare professional setting (adapted RUSH-3) according to gender, because of violation of the assumption of normality for these variables.

#### Self-reported training needs across profession

Chi-square test of independence was conducted to examine the association between the participants’ training needs (yes, no, in the future, other) and profession. Additionally, Fisher’s exact tests were used to assess the association between profession and intended provider of AI training.

### Ethical considerations

The present project followed the principles of the Declaration of Helsinki, and the recommendations of the Canada Tri-Council Policy Statement regarding research involving human participants. This project was approved by the Research Committee for sectorial research in neuroscience and mental health of the “CIUSSS Capitale Nationale” (approbation number: #2024–3079).

## Results

### Participants’ characteristics comparisons

A total of 114 hearing health professionals (51 audiologists, 63 hearing-aid acousticians) completed the online questionnaire (see Table 1 for a complete description of socio-professional characteristics). Respondents were aged between 21 and 66 years old (audiologists: 37.26 ± 1.34 years; hearing-aid acousticians: 38.83 ± 1.44 years) and age did not differ between professions (*U* = 1481, *p* = .476). All participants reported French as their native language, except four who identified it as a second language. Nonetheless, they had sufficient proficiency to complete the questionnaire, as provincial law requires them to provide services in French. Although women represented 75.45% (*n* = 86) of the total sample, significant differences between professions were identified for gender, with hearing-aid acousticians having a higher proportion of men (χ^2^ (1) = 5.848, *p* = .017). Gender discrepancies were determined to be representative of the provinces’ hearing health professionals’ population (χ^2^ (1) = 0.054, *p* = .816 for audiologists; χ^2^ (1) = 0.073, *p* = .787 for hearing-aid acousticians) [33–34]. No significant differences were observed between professions for the following variables: professional experience (*U* = 1601.500, *p* = .980), adoption of telepractice (χ^2^ (1) = 1.619, *p* = .244) and location of clinical practice (χ^2^ (2) = 0.518, *p* = .772). Furthermore, there were no significant differences in the location of clinical practice (χ^2^ (2) = 0.825, *p* = .662), personal use of AI (χ^2^ (1) = 1.788, *p* = .223) and professional use of AI (χ^2^ (1) = 0.946, *p* = .357) between professionals who do and do not engage in telepractice.

**Table 1:** Socio-professional characteristics

Variable	Level	Count (%)			*p*-value
		Audiologists *n* = 51	Hearing-aid acousticians *n* = 63	Total *n* = 114	
Gender					0.017^a^
	Men	7 (13.73)	21 (33.33)	28 (24.56)	
	Women	44 (86.27)	42 (66.67)	86 (75.44)	
Age					0.476^b^
	20–29	13 (25.49)	17 (26.98)	30 (26.32)	
	30–39	19 (37.25)	16 (25.40)	35 (30.70)	
	40–49	13 (25.49)	19 (30.16)	32 (28.07)	
	50–59	5 (9.80)	9 (14.29)	14 (12.28)	
	60 or above	1 (1.96)	2 (3.17)	3 (2.63)	
Professional experience					0.980^b^
	0–5	15 (29.41)	19 (30.16)	34 (29.82)	
	6–10	9 (17.65)	16 (25.40)	25 (21.93)	
	11–15	10 (19.61)	7 (11.11)	17 (14.91)	
	16–20	9 (17.65)	6 (9.52)	15 (13.16)	
	21–25	2 (3.92)	5 (7.94)	7 (6.14)	
	26 or above	6 (11.76)	10 (15.87)	16 (14.04)	
Location					
	Urban	29 (56.86)	38 (60.32)	67 (58.77)	
	Rural	14 (27.45)	18 (28.57)	32 (28.07)	
	Both	8 (15.69)	7 (11.11)	15 (13.16)	
Work setting					
	Private practice	20 (39.22)	63 (100.00)	83 (72.81)	
	Hospital/public clinic	23 (45.10)	-	23 (20.18)	
	Rehabilitation center	16 (31.37)	-	16 (14.04)	
	Industry	2 (3.92)	-	2 (1.75)	
	Teaching	5 (9.80)	1 (1.59)	6 (5.26)	
	Administrative	1 (1.96)	1 (1.59)	2 (1.75)	
	Other	2 (3.92)	-	2 (1.75)	
Clientele					
	Pediatric (0–5)	36 (70.59)	46 (73.02)	82 (71.93)	
	Pediatric (6–18)	43 (84.31)	51 (80.95)	94 (82.46)	
	Adult	46 (90.20)	63 (100.00)	109 (95.61)	
	Older adult	42 (82.35)	59 (93.65)	101 (88.60)	
Telepractice					0.244^a^
	Yes	13 (25.49)	10 (15.87)	23 (20.18)	
	No	38 (74.51)	53 (84.13)	91 (79.82)	
Personal use of AI					0.006^c^
	Yes	21 (41.18)	40 (63.49)	61 (53.51)	
	No	26 (50.98)	14 (22.22)	40 (35.09)	
	Uncertain	4 (7.84)	9 (14.29)	13 (11.40)	
Professional use of AI					< 0.001^a^
	Yes	14 (28.57)	49 (77.78)	63 (56.25)	
	No	35 (71.43)	14 (22.22)	49 (43.75)	

^a^ Fisher’s exact test

^b^ Mann-Whitney U-test

^c^ Chi-square test

As anticipated, significant differences were observed between professions in their reported fields of practice. Audiologists reported working more frequently than hearing-aid acousticians with individuals presenting the following conditions: tinnitus (χ^2^ (1) = 11.893, *p* < .001), eustachian tube dysfunction (χ^2^ (1) = 47.787, *p* < .001), hyperacusis/misophonia (χ^2^ (1) = 17.633, *p* < .001), auditory processing disorder (χ^2^ (1) = 14.370, *p* < .001), and vestibular disorders (χ^2^ (1) = 18.126, *p* < .001). Hearing-aid acousticians reported working more frequently with deaf blind individuals (χ^2^ (1) = 9.471, *p* = .003). Both professions had similar reported prevalence of work with individuals with noise-induced hearing loss (χ^2^ (1) = 2.318, *p* = .196).

Regarding prevalence of work with assistive and protective hearing devices in their professional practice, audiologists more often reported to work with assistive listening devices (χ^2^ (1) = 4.678, *p* = .046), cochlear implants (χ^2^ (1) = 10.037, *p* = .002), and bone-anchored hearing aids (χ^2^ (1) = 4.968, *p* = .033), while hearing-aid acousticians reported working with molded earplugs more often (χ^2^ (1) = 98.395, *p* < .001). For professional acts, auditory training (χ^2^ (1) = 4.888, *p* = .035), communication strategies (χ^2^ (1) = 12.936, *p* < .001), hearing aid recommendation (χ^2^ (1) = 7.544, *p* = .010) and educational audiology (χ^2^ (1) = 16.716, *p* < .001) were significantly more prevalently reported among audiologists while hearing aid sale (χ^2^ (1) = 114.000 *p* < .001) and hearing aid fitting/adjustments (χ^2^ (1) = 91.326, *p* < .001) were more often reported among hearing-aid acousticians. No differences across professions were found for cerumen removal (χ^2^ (1) = 2.312, *p* = .180). Finally, audiologists reported using more often these clinical assessments : otoacoustic emissions (χ^2^ (1) = 52.597, *p* < .001) and auditory evoked potentials (χ^2^ (1) = 13.541, *p* < .001), while hearing-aid acousticians reported doing more often hearing screening (χ^2^ (1) = 56.861, *p* < .001).

### Use of AI

More than half of respondents (*n* = 63) reported using AI in a professional setting. When asked how they used AI in their practice, half of the responses (51.26%) referred to its integration in hearing aids and AI powered programming. A fifth of AI application was related to text composition and editing, including emails, social media posts, assessment reports or advertisements. Some responses mentioned using AI to create or modify content (9.41%), such as images, audio recordings, auditory training activities or educational/pedagogical content. While some described using AI as a research and summary tool (8.24%), others mentioned AI use in hearing aid manufacturers’ mobile applications (4.71%). Few responses identified AI as a tool for data logging for hearing aid users (2.35%), translation (2.35%) and voice transcription (1.18%). Hearing-aid acousticians reported a higher likelihood of use of AI than their audiologist counterparts in both personal (χ^2^ (1) = 9.076, *p* = .003) and professional context (χ^2^ (1) = 27.118, *p* < .001). Whatever the profession, professionals using AI in their personal lives were more likely to use the technology in a professional setting (χ^2^ (1) = 27.492, *p* < .001).

### eHeatlh literacy skills

**Fig. 1:**
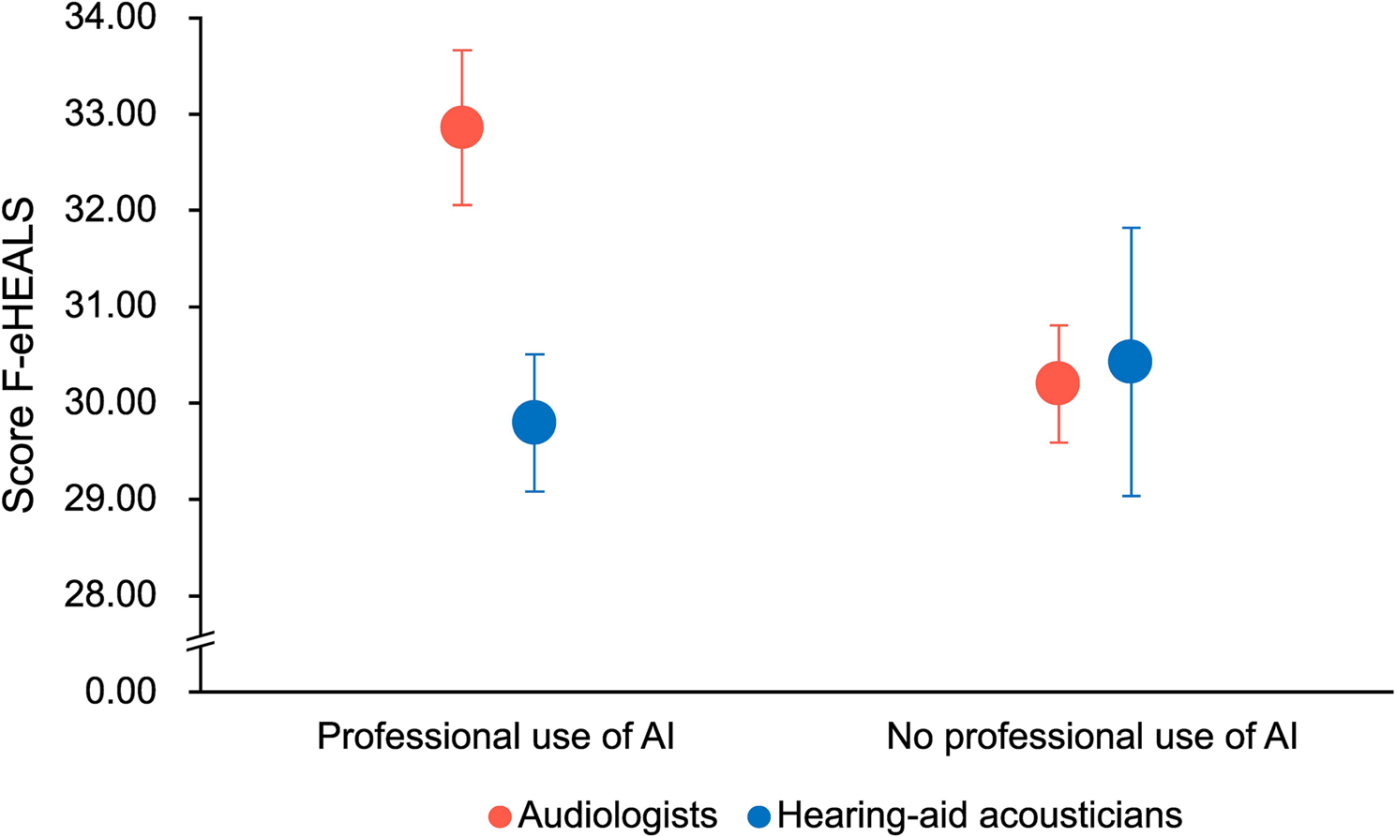
Scores on the French version of the eHeatlh Literacy Scale (F-eHEALS). Mean scores (±SEM) for each profession and professional use of AI are presented

The ART ANOVA revealed no significant main effect of profession (*F*(1,108) = 0.11, *p* = .740), professional use of AI (*F*(1,108) < 0.01, *p* = .999), nor a significant interaction effect (*F*(1,108) = 2.36, *p* = .127) on eHealth literacy scores (see Table 2 for detailed scores; Fig. 1). Additionally, there were no significant differences in eHealth literacy scores based on age (*H*(4) = 2.892, *p* = .576), professional experience (*H*(5) = 3.118, *p* = .682), clinical practice location (*H*(2) = 1.183, *p* = .554), adoption of telepractice (*U* = 981, *p* = .644), or personal use of AI (*U* = 1120, *p* = .487).

**Table 2:** Questionnaire scores

Variable	Profession		Professional use of AI		Overall score *n* = 114	Score per item *n* = 114
Questionnaire	Audiologists *n* = 51	Hearing-aid acousticians *n* = 63	Yes *n* = 63	No *n* = 49		
F-eHEALS	31.24 (0.53)	29.94 (0.63)	30.48 (0.60)	30.27 (0.58)	30.52 (0.42)	3.82 (0.05)
SHAIP	22.80 (0.55)	24.83 (0.45)	25.22 (0.46)	22.12 (0.49)	23.92 (0.36)	2.99 (0.05)
Perception of professional impact	17.08 (0.45)	17.57 (0.33)	17.92 (0.35)	16.49 (0.40)	17.35 (0.27)	3.47 (0.05)
Perception of preparedness for AI	5.73 (0.23)	7.25 (0.25)	7.30 (0.25)	5.63 (0.24)	6.57 (0.19)	2.19 (0.06)
AISES	89.18 (1.78)	95.49 (1.90)	96.30 (1.78)	87.33 (1.87)	92.67 (1.35)	4.21 (0.06)
Assistance	34.20 (0.79)	36.02 (0.67)	36.91 (0.59)	32.59 (0.75)	35.20 (0.52)	5.03 (0.07)
Anthropomorphic interaction	13.31 (0.59)	15.05 (0.67)	14.46 (0.65)	14.18 (0.65)	14.27 (0.46)	2.85 (0.09)
Comfort with AI	24.39 (0.78)	25.79 (0.87)	26.41 (0.78)	23.31 (0.88)	25.17 (0.59)	4.19 (0.10)
Technological skills	17.28 (0.65)	18.64 (0.57)	18.52 (0.57)	17.25 (0.67)	18.03 (0.43)	4.51 (0.11)
Adapted RUSH-6	22.08 (0.46)	23.73 (0.38)	23.98 (0.34)	21.67 (0.49)	22.99 (0.30)	3.83 (0.05)
Adapted RUSH-3	12.02 (0.28)	12.62 (0.22)	12.81 (0.22)	11.74 (0.27)	12.35 (0.18)	4.12 (0.06)

Scores presented as *M* (±SEM)

### Evaluation of artificial intelligence perception

**Fig. 2:**
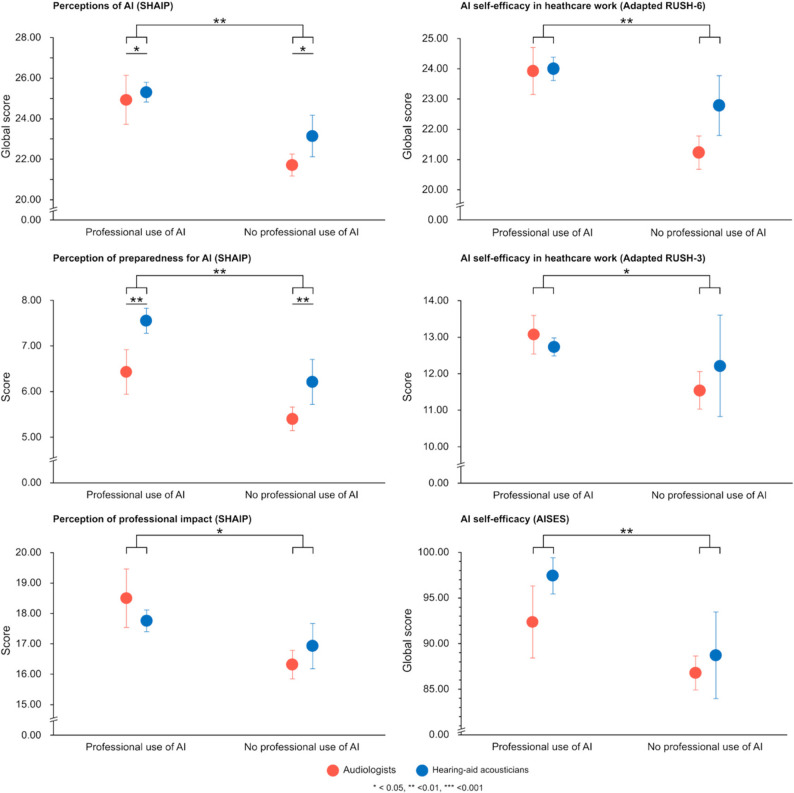
Scores for perceptions of AI (SHAIP), AI self-efficacy in healthcare work (adapted versions of RUSH) and AI self-efficacy (AISES). Mean scores (±SEM) for each profession and professional use of AI are presented. For SHAIP, global scores and scores to both dimensions are presented. For the adapted RUSH, both global and subset scores are presented. For the AISES, global scores are presented

The MANOVA revealed significant main effect of profession (*Wilks’ Lambda* = 0.944, *F*(1,107) = 3.192, *p* = .045, partial η^2^ = 0.056) and professional use of AI (*Wilks’ Lambda* = 0.892, *F*(1,107) = 6.447, *p* = .002, partial η^2^ = 0.108). Moreover, the interaction effect between profession and professional use of AI was not significant (*Wilks’ Lambda* = 0.985, *F*(2,107) = 0.797, *p* = .453), indicating that the combined influence of these variables did not significantly impact joined *perception of professional impact* and *perception of preparedness for AI* (see Table 2 for detailed scores; Fig. 2).

Univariate tests for between subject effects were conducted to further examine the individual effects on *perception of professional impact* and *perception of preparedness for AI.* Professional use of AI had significant effects on both *perception of professional impact* (*F*(1,108) = 6.117, *p* = .015, partial η^2^ = 0.054) and *perception of preparedness for AI* (*F*(1,108) = 9.148, *p* = .003, partial η^2^ = 0.078). Furthermore, profession significantly influenced the results obtained on *perception of preparedness for AI* (*F*(1,108) = 6.133, *p* = .015, partial η^2^ = 0.054), with hearing-aid acousticians scoring significantly higher than audiologists. Profession did not however have significant influence on *perception of professional impact* (*F*(1,108) = 0.012, *p* = .915). Additionally, global scores were also significantly higher for participants who were using AI in their personal lives (t(99) = 3.429, *p* < .001). There were no significant differences in perceptions of AI scores based on age (*F*(4,109) = 0.475, *p* = .754), professional experience (*F*(5,108) = 0.374, *p* = .865), location of clinical practice (*F*(2,111) = 0.959, *p* = .387) and adoption of telepractice (t(112) = 0.109, *p* = .913).

### General AI self-efficacy

The MANOVA revealed significant main effect of professional use of AI (*Wilks’ Lambda* = 0.854, *F*(4,105) = 4.485, *p* = .002, partial η^2^ = 0.146). However, there was no significant main effect of profession (*Wilks’ Lambda* = 0.950, *F*(4,105) = 1.370, *p* = .249) on hearing health professionals’ general AI self-efficacy. There was also no significant interaction effect between profession and professional use of AI (*Wilks’ Lambda* = 0.990, *F*(4,105) = 0.270, *p* = .897) on hearing health professionals’ general AI self-efficacy, indicating that the combined influence of these variables did not significantly impact pooled score of *assistance*, *anthropomorphic interaction*, *comfort with AI*, and *technological skills* (see Table 2 for detailed scores; Fig. 2).

Univariate tests for between subject effects were conducted to further examine the individual effects on *assistance*, *anthropomorphic interaction*, *comfort with AI* and *technological skills*. Professional use of AI had significant effects on both *assistance* (*F*(1,108) = 14.801, *p* < .001, partial η^2^ = 0.121) and *comfort with AI* (*F*(1,108) = 4.698, *p* = .032, partial η^2^ = 0.042), but not on *anthropomorphic interaction* (*F*(1,108) = 0.423, *p* = .517) and *technological skills* (*F*(1,108) = 0.467, *p* = .496). Additionally, global scores were also significantly higher for professionals who are using AI in their personal lives (*U* = 559.500, *p* < .001). There were no significant differences on general AI self-efficacy scores based on age (*F*(4,109) = 0.541, *p* = .706), professional experience (*F*(5,108) = 1.156, *p* = .336), location of clinical practice (*F*(2,111) = 2.908, *p* = .059) and adoption of telepractice (t(112) = 1.799, *p* = .075).

### AI self-efficacy in a healthcare professional setting

The ANOVA revealed significant main effect of professional use of AI (*F*(1,108) = 8.630, *p* = .004, partial η^2^ = 0.074) on hearing health professionals’ AI self-efficacy in healthcare work (see Table 2 for detailed scores; Fig. 2). However, there was no significant main effect of profession (*F*(1,108) = 1.494, *p* = .224) on hearing health professionals’ AI self-efficacy in a healthcare professional setting. There was also no significant interaction effect between profession and professional use of AI (*F*(1,108) = 1.243, *p* = .267) on hearing health professionals’ AI self-efficacy in healthcare work. Additionally, scores were significantly higher for professionals who are using AI in their personal lives (t(99) = 3.456, *p* < .001). There were no significant differences on AI self-efficacy in a healthcare professional setting scores based on age (*F*(4,109) = 0.561, *p* = .692), professional experience (*F*(5,108) = 0.908, *p* = .479), location of clinical practice (*F*(2,111) = 2.998, *p* = .054) and adoption of telepractice (t(112) = 1.175, *p* = .243).

The subset score of the adapted RUSH-3 showed similar results. The ANOVA revealed significant main effect of professional use of AI (*F*(1,108) = 6.538, *p* = .012, partial η^2^ = 0.057) on hearing health professionals’ AI self-efficacy in healthcare work (see Table 2 for detailed scores; Fig. 2). However, there was no significant main effect of profession (*F*(1,108) = 0.174, *p* = .677) on hearing health professionals’ AI self-efficacy in a healthcare professional setting. There was also no significant interaction effect between profession and professional use of (*F*(1,108) = 1.583, *p* = .211) on hearing health professionals’ AI self-efficacy in healthcare work. Additionnally, global scores were also significantly higher for participants using AI in their personal lives (*U* = 820.500, *p* = .005). As for location, professionals practicing in urban areas versus those in a mixed urban/rural practice had significantly higher scores (*W* = 6.019, *p* = .049). There were no significant differences on AI self-efficacy in a healthcare professional setting scores based on age (*F*(4,109) = 1.349, *p* = .256), professional experience (*F*(5,108) = 1.031, *p* = .403), and adoption of telepractice (*U* = 880, *p* = .234).

### Influence of gender on outcome measures

Investigations revealed no significant differences across gender for eHealth literacy (U = 942, *p* = .083), perceptions of AI (t(112) = 1.659, *p* = .100), or AI self-efficacy in healthcare work (RUSH-6; t(112) = 1.788, *p* = .076). Interestingly, significant differences were found for the RUSH-3 subset of AI self-efficacy in healthcare work, with men scoring higher than women (U = 783.500, *p* = .005). Furthermore, significant gender differences were also observed in general AI self-efficacy, with men reporting higher overall scores (t(112) = 2.931, *p* = .004).

By examining the relationship across study variables, significant positive correlations were found between eHealth literacy skills and perceptions of AI (*r*_*s*_ = 0.185, *p* = .048) and between eHealth literacy skills and AI self-efficacy in a healthcare setting (*r*_*s*_ = 0.320, *p* < .001). The perceptions of AI was positively correlated to general AI self-efficacy (*r*_*s*_ = 0.410, *p* < .001) and to AI self-efficacy in a healthcare setting (*r*_*s*_ = 0.369, *p* < .001). Finally, general AI self-efficacy was positively correlated to AI self-efficacy in a healthcare setting (*r*_*s*_ = 0.515, *p* < .001).

### Training needs

**Fig. 3:**
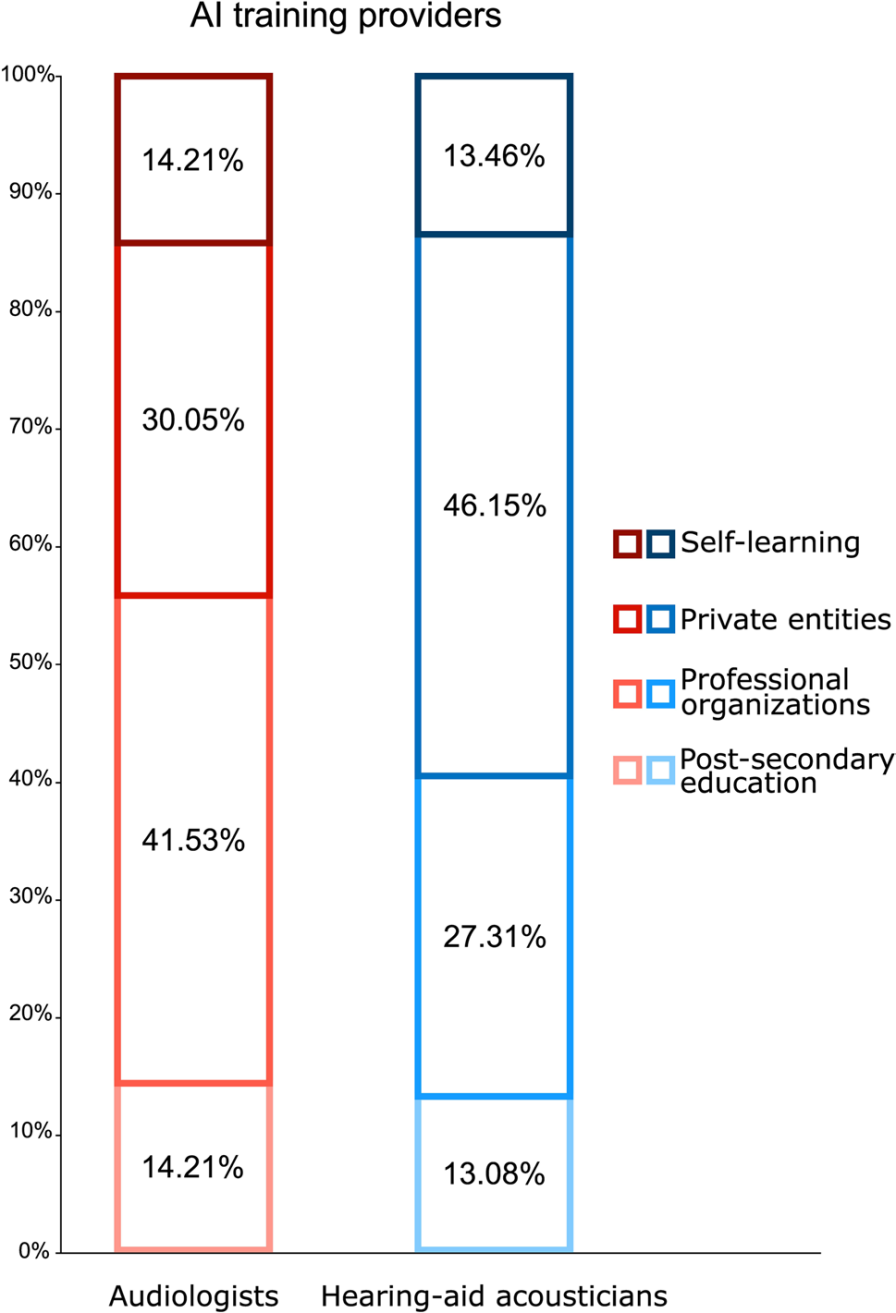
Preferred AI training providers identified among each profession. Percentages represent the proportion of responses selected for each AI training provider, by profession

**Fig. 4:**
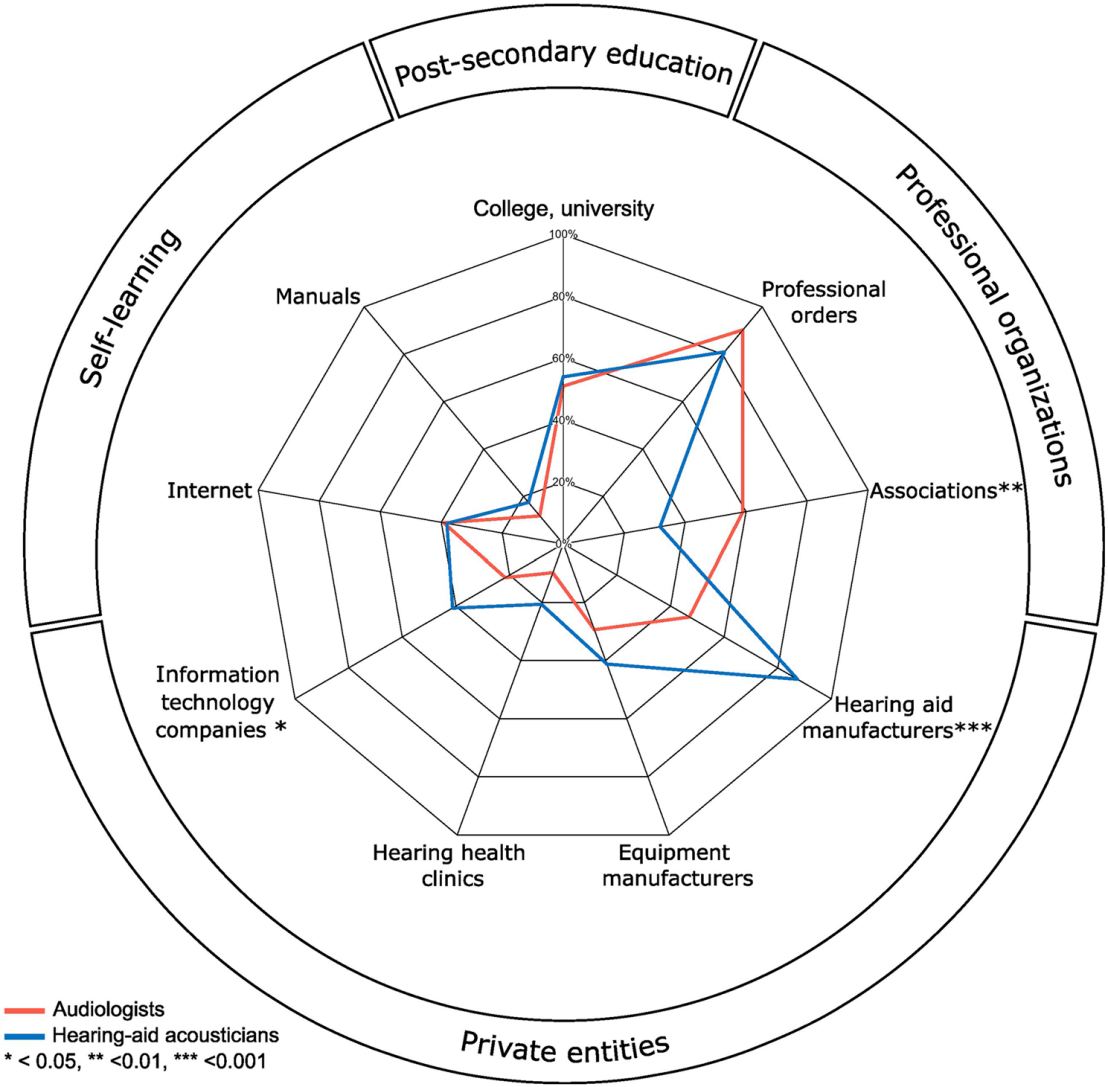
Comparison between profession for preferred AI training provider. Percentages represent the proportion of participants, by profession, who selected each AI training provider

A majority of hearing health professionals (71.05%) reported a need to obtain further training on the use of AI in clinical settings, with 68.63% of audiologists and 60.32% of hearing-aid acousticians expressing a desire for imminent training. An additional 7.84% of audiologists and 6.35% of hearing-aid acousticians foresee needing AI training in the future. No significant differences between professions were found regarding training needs (χ^2^ (3) = 3.536, *p* = .316). When asked which entities should be responsible for providing such training, the two most commonly reported were private entities (audiologists: 30.05%; hearing-aid acousticians: 46.15%), and professional organizations (audiologists: 41.53%; hearing-aid acousticians: 27.31%, Fig. 3). However, audiologists selected professional associations significantly more than hearing-aid acousticians (χ^2^ (1) = 8.392, *p* = .005) while the latter significantly selected hearing aid manufacturers (χ^2^ (1) = 21.453, *p* < .001) and information technology companies (χ^2^ (1) = 4.990, *p* = .029) over audiologists (Fig. 4).

## Discussion

The main goal of this study was to examine hearing health professionals’ eHealth literacy skills, perceptions of AI and perceived AI self-efficacy as a proxy to document their comfort with AI-based technologies. As such, we assessed whether profession, professional use of AI or their interaction were associated with hearing health professionals’ eHealth literacy skills, perceptions of AI and AI self-efficacy. Our results show no interaction effects of profession and professional use of AI for each of these constructs. Although, eHealth literacy skills were found to be similar across both profession and professional use of AI, the perceptions of AI were determined to be more positive for hearing-aid acousticians and for professionals who are using AI in a professional setting. Additionally, general AI self-efficacy and AI self-efficacy in a healthcare setting were higher for professionals who are using the technology in a professional setting, regardless of their profession. Our results highlight the positive relationships between the constructs under investigation, where hearing health professionals with higher eHealth literacy skills were related to more positive perceptions of AI and a higher AI self-efficacy in a healthcare setting. The perceptions of AI were also found to be more positive for hearing health professionals with higher AI self-efficacy. Finally, a higher general AI self-efficacy is associated to a higher AI self-efficacy in a healthcare setting. Interestingly, when examining the potential effect of gender on the constructs under investigation, the only differences between genders were on AI self efficacy, which was seemingly higher in men hearing health professionals in comparison to their women colleagues. Lastly, while majority of hearing health professionals reported desires for AI training, each profession identified difference source of training. Audiologists were more likely to identify professional order as their desired source of training while hearing-aid acousticians selected the corporate sector to provide such training.

### Methodological challenges in assessing health professional AI capabilities

A key takeaway from this study concerns the evaluation tools used in the integration of AI by healthcare professionals and within healthcare settings, particularly highlighting the lack of interoperability among these tools. Despite the growing importance that current and future AI-powered applications (either disembodied or embodied) could have to answer to the shortage of health professionals [44, 45, 6], evaluation of health professionals capabilities to deal adequately with AI is still made difficult by the relative paucity of unified and validated evaluation tools. While true for the present study, this limitation does apply to any study attempting to evaluate the perception and integration capabilities of AI by health professionals. Indeed, the main challenge which was faced when designing this study was not to recruit participants, but to select a proper set of evaluation tools that would allow to optimally seize these professionals’ capabilities of AI integration.

Interestingly, the paucity of tools does not imply a complete absence of validated questionnaires. Some tools (such as the ones selected in the present study) are still adequate to use to evaluate healthcare professionals’ attitudes toward AI [41]. There are however several limitations to their optimal utilization. First, in the emerging context of AI, the concepts being evaluated are often not operationalized fully. Second, most of the existing tools are evaluating very specific constructs/questions, thus requiring some adaptation across studies. For instance, this was the case with the RUSH scale, which, although originally developed to assess technology use in healthcare, was specifically designed for robots and had to be adapted to evaluate the same construct in the context of AI [43]. Third, the recency of the problematic in the literature – and thus of the evaluation tools – makes the tools unlikely to be available in languages other than English. While translations can easily be done (especially given the short length of most of these tools), translated versions are typically not systematically validated with a broad population compared to the original versions. Interestingly, in contrast to a lot of classical psychometric tools, it should however be noted that original versions of AI-related scales are usually not validated on large populations either. Fourth, the diversity of possible dimensions coupled with the specificity of the current tools makes the need for triangulation prominent, requiring researchers to combine several evaluation tools to properly approximate participants’ AI capabilities (in the present study, a total of four different validated tools were used in addition to the socio-professional questionnaire). Finally, the question of control and baseline of technology savviness, which could be an obvious confounding factor, is usually not addressed by current tools. Indeed, available AI scales typically solely explore attitudes to, familiarity with, or ease of use of AI without considering the technology as a whole – with the noticeable exception of the AISES, which was used in the present study [42]. In the present study, this concern was further addressed by using eHealth literacy as a proxy for medical technology familiarity. However, this approach is suboptimal for promoting replication, and eHealth literacy should ideally be directly incorporated into AI-related questionnaires and not be evaluated separately. Consequently, current health professionals’ AI perception research suffers from a major issue of interoperability, pinpointing the need to develop and validate standardized tools for assessing use of AI in the medical field.

### Diversity of perception of AI across hearing health professionals

The eHealth Literacy Scale has been widely used in the literature to evaluate eHealth literacy across various populations [37, 46–47]. One finding from this study is that both hearing-aid acousticians and audiologists presented equivalent levels of eHealth literacy. Surprisingly, and despite its ease of use, this tool has only seldomly been used to explore healthcare professionals’ eHealth literacy skills [48]. However, our results are comparable with to those observed in a sample of 149 German healthcare professionals (including nurses, psychologists, physical therapists, physicians, occupational therapists) who reported a mean score of 31.84 compared to the 30.52 found in our study [49]. In a similar vein, the hearing health professionals in our sample demonstrated scores comparable to those of Canadian students enrolled in a pharmacy doctoral program, who achieved a mean score of 31.07 [50]. Of note, while such levels of eHealth literacy could be considered relatively high, they do not represent the upper limit, even among healthcare professionals. Indeed, those with greater exposure to information technologies may demonstrate even higher scores [51]. This is for instance the case of neonatal nurses – a subspecialty of nurses heavily relying upon technology through intensive use of electronic medical records, computerized interfaces, and advanced technological patient monitoring – who displayed higher scores than our sample (mean per item of 4.32 vs. 3.81 respectively) [51]. From this perspective, the population investigated in our study can really be considered as representative of a “standard” health professional population.

Moreover, results from this study concerning hearing health professionals’ perceptions of AI was also similar to what has been seen for other healthcare professionals when considering the professional impact of AI. Similar scores were observed between our sample and a large population of various clinical and non-clinical health workers [41] including notably speech pathology, podiatry, physiotherapy, social work, occupational therapy (mean per item of 3.48 vs. 3.47 in our sample for the professional impact of AI, and 2.29 vs. 2.19 in our sample for the preparedness for AI), and also between our sample and a large cohort of nurse leaders (mean per item of 3.72 vs. 3.47 in our sample for the professional impact of AI, and slightly lower for preparedness for AI with 3.32 vs. 2.19 in our sample) [52].

Although hearing health professionals expressed more positive views of AI as an assistive tool and adequate technological skills, their overall comfort with the technology could still be improved. Indeed, the hearing health professionals surveyed in this study presented relatively low levels of general AI self-efficacy, with scores placing them slightly above the 20th percentile for the hearing-aid acousticians and slightly above the 10th percentile for the audiologists, when compared to the norms proposed by the initial developers of the scale [42]. It should however be noted here that, while extremely interesting when it comes to the constructs evaluated, the AISES has yet to be validated across large populations to ensure the representativeness of the distribution of the responses. From this perspective, one of the few studies so far having used the complete AISES questionnaire to explore general AI self-efficacy found very similar scores (mean per item of 4.51 vs. 4.21 in our study) with another, unrelated population – in this case 429 university students enrolled in an English as a second language class [53]. However, the very low scores measured among hearing health professionals from this study in the anthropomorphic interaction dimension could be an indicator that, in the context of hearing health, interacting with a disembodied artificial agent would not feel as natural as interacting with a Human entity. Hearing health professionals’ AI self-efficacy in a healthcare setting was similar to what other healthcare professionals displayed when using healthcare robots, as evaluated either by the RUSH-6 with both Finish home care workers (mean per item of 3.54, *n* = 100) [54] or Chinese nurses (3.58, *n* = 368) [55] displaying scores comparable to those seen here (3.83, in our version adapted for AI instead of robots), or by the RUSH-3 with the same limits, for instance with a large sample of Finish home care workers (mean per item of 4.19, *n* = 3377, compared to 4.12 in our sample [56].

Interestingly, this study puts forward varied attitudes from hearing health professionals towards the use of AI in a clinical setting. In general, our results show that higher eHealth literacy skills are correlated to more positive perceptions of AI and higher AI self-efficacy in the workplace. While differences were found in the perception of preparedness for AI between profession, most of the differences identified in terms of perceptions of AI, general and workplace self-efficacy were explained by the use of AI in a professional setting. In fact, differences could not be explained by age, professional experience, or location of clinical practice. Surprisingly, the adoption of telepractice was not a factor which could explain these differences. The differences in AI self-efficacy between genders (as attested by the results of AISES and RUSH-3) could potentially be explained by the fact that men hearing health professionals tend to use AI, both in their personal and professional lives, more than their women counterparts. Professionals with higher general AI self-efficacy skills usually also have higher AI self-efficacy in a healthcare setting. Hearing health professionals with higher general and work-related AI self-efficacy skills tend to have a more positive perception of the technology. However, for similar professions in a shared field of practice, the differences found could be attributed to prior use of AI in a professional setting over the profession itself.

### Going beyond acknowledging the needs for training

Perception of AI and self-efficacy with AI of hearing health professionals is directly related to the use of AI tools in a professional setting. Therefore, training these professionals on the functioning of these new technologies would be important in order to ensure the proper use of these technologies, and to propel positive outcomes [45]. Indeed, understanding how AI works and what it does within an intelligent hearing device would allow hearing health professionals to optimize their recommendations relative to the use that patients can make of them. Of note, the importance of AI-powered tools for hearing health clinical decision support has been self-acknowledged by hearing health professionals in other surveys [25].

However, AI could find its application beyond the utilization within therapeutic devices (something obviously prominent in the context of hearing health), notably during the Human-centered cognitive processes. Indeed, in a context of AI-assisted decision-making for health professionals, AI triage recommendations have been shown to be congruent with expert judgments of psychologists [57]. Therefore, providing adequate training to hearing health professionals to increase their skills using AI tools would potentially improve the quality of the therapeutic process, as suggested for hearing health professionals [25] and for other health professions alike [45].

The call for increased AI training among healthcare professionals has gained momentum since the emergence of large language models (LLMs), with several proposals already put forward in this direction [58], notably for mental healthcare professionals [45]. Our results, showing that hearing health professionals reported a need for AI-related training, are in line with such needs reported in other hearing health professional populations, such as ENT specialists and audiologists [25]. One could think that, ideally, such should be sourced by a formal inclusion of AI training into health professionals’ academic curricula. However, in the present study, post-secondary education institutions were not the source of formation selected the most by hearing health professionals as a source of AI training. Contrary to expectations audiologists massively selected professional organizations as the preferred primary source for providing AI training, while hearing-aid acousticians massively selected private entities – specifically hearing aid manufacturers – alongside professional organizations as their main choices. Post-secondary institutions were selected as the preferred providers of AI training by only half of the respondents. Several factors could contribute to explaining this relatively low rate of occurrence. This situation could be related to the perception by clinicians of the adequation of the post-secondary programs with the evolving professional reality. In any case, this question should be investigated further. Alternatively, the fact that, in the context of hearing health professionals, most of the post-graduate training is being done by professional organizations could have contributed to these professionals identifying them as the logical source for further professional training. Yet, this raises the question of access to expertise. Indeed, while universities do have direct access to a wide set of skills, competencies, and expertise, professional orders typically have access only to the expertise of the profession they primarily represent. Of course, AI expertise could be gathered externally, but such a strategy would bear significant risks regarding the control of the quality of the training delivered (a problem that would be less prominent in a context of a university, for instance). If the independence of professional organizations can be considered equivalent to post-secondary education programs, it is unlikely to be the case for manufacturers. The AI training they provide will inevitably have bias, as they ultimately want to sell their products to professionals.

## Conclusions

While being already extremely promising, AI is nonetheless an emerging technology, and significant developments can be expected in the near future. In this view, perceptions of AI are continuously evolving and could change significantly in a short period of time, due notably (in a hearing health professional context) to the exposure to new products or available training. As mentioned earlier, AI in the context of hearing health can have applications going well beyond the augmentation of hearing aids’ capabilities. In the age of big data, the capacity of AI to gather a considerable amount of information related to the patients’ health situation will lead to discussions and debates related to the accessibility of these data, and the real forms of informed consent [59]. If only marginally mentioned in our sample, ethical concerns have been reported by hearing health professionals regarding the future use of AI [25]. Thus, optimal training cycles on AI designed for hearing health professionals should cover, besides purely technical aspects, ethical considerations of the use of AI in a healthcare setting, notably including questions related to patient safety, data security, professional responsibility, accessibility to technology, or AI biases. In this context full of challenges, the clear openness of the hearing health professionals to these technologies evidenced in the present study, is a beacon of hope for the future of hearing health in an age of technology.

## Electronic Supplementary Material

Below is the link to the electronic supplementary material.

Supplementary Material 1

## Data Availability

The datasets generated and/or analyzed during the current study are available from the corresponding author on reasonable request.

## Abbreviations

AI: Artificial intelligence
AISES: Artificial Intelligence Self-Efficacy Scale
ART ANOVA: Aligned Rank Transform Analysis of Variance
eHEALS: eHealth Literacy Scale
F-eHEALS: French version of the eHealth Literacy Scale
MANOVA: Multivariate Analysis of Variance
RUSH: Robot Use Self-Efficacy in Healthcare Work
SEM: Standard error of the mean
SHAIP: Shinners Artificial Intelligence Perception Tool
