# Supplementary material for: Hearing health professionals’ attitudes and perceived skills toward artificial intelligence
Source: BMC Med Educ. 2026 Jan 10;26:218. doi: 10.1186/s12909-025-08505-9 (PMC12882145; doi:10.1186/s12909-025-08505-9)
Supplement: Supplementary file 1 — Supplementary Material 1 [file 12909_2025_8505_MOESM1_ESM.docx]

| **Section 1** |
| --- |
| 1. Age : |
| 1. Gender [Select all applicable] :  Agender  Bigender/multigender  Two-Spirit  Questioning  Woman  Gender fluid  Gender queer  Man  Non-binary  Transgender  Prefer not to say  Other gender identity (specify) : |
| 1. Native language : |
| 1. Profession :  Audiologist  Hearing-aid acoustician  Other, specify : |
| 1. Age at graduation : |
| 1. Membership to a professional order  Ordre des orthophonistes et des audiologistes du Québec  Ordre des audioprothésistes du Québec  Other, specify : |
| 1. Experience as a hearing health professional (years) : |
| 1. Location of clinical practice :  Rural  Urban |
| 1. Work setting [Select all applicable]:  Private practice  Hospital/public clinic  Rehabilitation center  Industry  Teaching  Administrative  Other, specify : |
| 1. Clientele [Select all applicable]:  Pediatric (0-5 years old)  Pediatric (6-18 years old)  Adult  Older adults |
| 1. Field of practice [Select all applicable]:  Tinnitus  Eustachian tube dysfunction  Hyperacusis/misophonia  Deaf blindness  Noise-induced hearing loss  Auditory processing disorder  Vestibular disorders  Assistive listening devices  Molded earplugs  Cochlear implants  Bone-anchored hearing aids  Auditory training  Cerumen removal  Communication strategies  Hearing aid recommendation  Hearing aid sale  Hearing aid fitting/adjustments  Educational audiology  Otoacoustic emissions  Auditory evoked potentials  Hearing screening  Other, specify : |
| 1. I use telepractice :  Yes  No |

| **Section 2** |
| --- |
| 1. I use artificial intelligence (AI) in my personal life :  Yes  No  Uncertain |
| 1. Do you currently use AI in a professional setting? If so, to what extent? What is your appreciation of its usage? |
| 1. Do you think you need more training regarding AI in hearing health? Explain what your training needs would be on the topic of AI in a clinical setting. |
| 16. In your opinion, who should provide this type of professional training? [Select all applicable]:  Post-secondary education (College, University)  Professional orders  Professional associations  Manufacturers (hearing aids, implants)  Equipment manufacturers  Hearing health clinics  Information technology compagnies  Self-learning through Internet  Self-learning through manuals |

| **Section 3** | | | | | |
| --- | --- | --- | --- | --- | --- |
| Questions | Not useful at all | Not useful | Unsure | Useful | Very Useful |
| 1. How **useful** do you feel the Internet is in helping you in making decisions about your health? | 1 | 2 | 3 | 4 | 5 |
|  | Not important at all | Not important | Unsure | Important | Very important |
| 1. How **important** is it for you to be able to access health resources on the Internet? | 1 | 2 | 3 | 4 | 5 |
|  | Strongly Disagree | Disagree | Undecided | Agree | Strongly Agree |
| 1. I know **what** health resources are available on the Internet. | 1 | 2 | 3 | 4 | 5 |
| 1. I know **where** to find helpful health resources on the Internet. | 1 | 2 | 3 | 4 | 5 |
| 1. I know **how** to find helpful health resources on the Internet. | 1 | 2 | 3 | 4 | 5 |
| 1. I know **how to use** the Internet to answer my questions about health. | 1 | 2 | 3 | 4 | 5 |
| 1. I know how to use **the health information** I find on the Internet to help me. | 1 | 2 | 3 | 4 | 5 |
| 1. I have the skills I need to **evaluate** the health resources I find on the Internet. | 1 | 2 | 3 | 4 | 5 |
| 1. I can tell **high quality** health resources from **low quality** health resources on the Internet. | 1 | 2 | 3 | 4 | 5 |
| 1. I feel **confident** in using information from the Internet to make health decisions. | 1 | 2 | 3 | 4 | 5 |

| **Section 4** | | | | | |
| --- | --- | --- | --- | --- | --- |
|  | Strongly Disagree | Disagree | Undecided | Agree | Strongly Agree |
| 1. I believe that the use of AI in my specialty could improve the delivery of patient care. | 1 | 2 | 3 | 4 | 5 |
| 1. I believe that the use of AI in my specialty could improve clinical decision making. | 1 | 2 | 3 | 4 | 5 |
| 1. I believe that AI can improve population health outcomes. | 1 | 2 | 3 | 4 | 5 |
| 1. I believe that AI will change my role as a healthcare professional in the future. | 1 | 2 | 3 | 4 | 5 |
| 1. I believe that the introduction of AI will reduce financial cost associated with my role. | 1 | 2 | 3 | 4 | 5 |
| 1. I believe that overall healthcare professionals are prepared for the introduction of AI technology. | 1 | 2 | 3 | 4 | 5 |
| 1. I believe that I have been adequately trained to use AI that is specific to my role. | 1 | 2 | 3 | 4 | 5 |
| 1. I believe there is an ethical framework in place for the use of AI technology in my workplace. | 1 | 2 | 3 | 4 | 5 |

| **Section 5** | | | | | |
| --- | --- | --- | --- | --- | --- |
|  | Totally Disagree | Disagree | Undecided | Agree | Totally Agree |
| 1. Generally speaking, I consider myself technologically competent. | 1 | 2 | 3 | 4 | 5 |
| 1. I’m confident in my ability to learn how to use artificial intelligence if it were to become part of my practice. | 1 | 2 | 3 | 4 | 5 |
| 1. I believe that it would be easy for me to learn how to use artificial intelligence in home care in the future. | 1 | 2 | 3 | 4 | 5 |
| 1. I’m confident in my ability to learn simple programming of artificial intelligence if I were provided the necessary training. | 1 | 2 | 3 | 4 | 5 |
| 1. I’m confident in my ability to learn how to use artificial intelligence in order to guide others to do the same. | 1 | 2 | 3 | 4 | 5 |
| 1. I believe that teaching elderly people how to use artificial intelligence would not be difficult for me. | 1 | 2 | 3 | 4 | 5 |

| **Section 6** | | | | | | | |
| --- | --- | --- | --- | --- | --- | --- | --- |
|  | Strongly Disagree | Disagree | Slightly Disagree | Undecided | Slightly Agree | Agree | Strongly Agree |
| 1. Some AI technologies/products make learning easier. | 1 | 2 | 3 | 4 | 5 | 6 | 7 |
| 1. I find that AI technologies/products are helpful for learning. | 1 | 2 | 3 | 4 | 5 | 6 | 7 |
| 1. AI technologies/products are good aids to learning. | 1 | 2 | 3 | 4 | 5 | 6 | 7 |
| 1. Using AI technologies/products makes learning more interesting. | 1 | 2 | 3 | 4 | 5 | 6 | 7 |
| 1. I’m confident in my ability to learn simple programming of AI technologies/products if I were provided the necessary training. | 1 | 2 | 3 | 4 | 5 | 6 | 7 |
| 1. AI technologies/products help me to save a lot of time. | 1 | 2 | 3 | 4 | 5 | 6 | 7 |
| 1. I find it easy to get AI technologies/products to do what I want it to do. | 1 | 2 | 3 | 4 | 5 | 6 | 7 |
| 1. I think the interactive process of AI technologies/products is very vivid, just like chatting with a real person. | 1 | 2 | 3 | 4 | 5 | 6 | 7 |
| 1. I think the way that AI technologies/products express content when interacting is unique, just like a real person. | 1 | 2 | 3 | 4 | 5 | 6 | 7 |
| 1. I think there is no difference between the dialogue method of AI technologies/products compared with the dialogue with real people. | 1 | 2 | 3 | 4 | 5 | 6 | 7 |
| 1. I think the tone of AI technologies/products when interacting is the same as that of real people. | 1 | 2 | 3 | 4 | 5 | 6 | 7 |
| 1. I feel that the way of expression of AI technologies/products in the interactive text is the same as that of real people. | 1 | 2 | 3 | 4 | 5 | 6 | 7 |
| 1. When interacting with AI technologies/products, I feel very calm. | 1 | 2 | 3 | 4 | 5 | 6 | 7 |
| 1. When interacting with AI technologies/products, I find it easy. | 1 | 2 | 3 | 4 | 5 | 6 | 7 |
|  | | | | | | | |
|  | | | | | | | |
|  | Strongly Disagree | Disagree | Slightly Disagree | Undecided | Slightly Agree | Agree | Strongly Agree |
| 1. When interacting with AI technologies/products, I feel comfortable in my heart. | 1 | 2 | 3 | 4 | 5 | 6 | 7 |
| 1. When interacting with AI technologies/products, I feel very peaceful. | 1 | 2 | 3 | 4 | 5 | 6 | 7 |
| 1. When interacting with AI technologies/products, I feel very relaxed. | 1 | 2 | 3 | 4 | 5 | 6 | 7 |
| 1. I can happily interact with AI technologies/products smoothly. | 1 | 2 | 3 | 4 | 5 | 6 | 7 |
| 1. When using AI technologies/products, I am not worried that I might press the wrong button and cause risks. | 1 | 2 | 3 | 4 | 5 | 6 | 7 |
| 1. When using AI technologies/products, I am not worried that I might press the wrong button and damage it. | 1 | 2 | 3 | 4 | 5 | 6 | 7 |
| 1. When using an AI technology/product, there is nothing that I do not know why. | 1 | 2 | 3 | 4 | 5 | 6 | 7 |
| 1. AI technologies/products jargon does not baffle me. | 1 | 2 | 3 | 4 | 5 | 6 | 7 |

| **Section 7** |
| --- |
| 63. Do you have any other comments regarding the use of AI in your profession? |
